# Cas rare de fracture des deux os de l’avant-bras chez un patient présentant une bifurcation de l'humérus et une déficience ulnaire: synostose huméro-radioulnaire

**DOI:** 10.11604/pamj.2022.41.316.34525

**Published:** 2022-04-20

**Authors:** Hicham Douma, El Hassani Abdelkrim

**Affiliations:** 1 Service de Traumatologie A, Hôpital Ibn Tofail, CHU Mohammed VI, Faculté de Médecine et de Pharmacie de Marrakech, Université Cadi Ayyad de Marrakech, Marrakech, Maroc

**Keywords:** Synostose, huméro-radioulnaire, fracture, Synostosis, humero-radioulnar, fracture

## Abstract

Humero-radioulnar synostosis is a rare deformity of the upper limb. The synostosis of the elbow is a malformation that may happen between any of the bones of this joint. The most common one is radioulnar synostosis. Humero-radioulnar synostosis has been very little reported in the literature. We report the case of a 32 years old man with a condition of phacomolia, consulting in the emergency room, for pain of the left upper limb after a light football trauma. The patient also had craniosynostosis of the lambdoid suture. Left-arm X-ray showed a bifurcating humerus, with ulnar deficiency, and non-displaced radial and ulnar fractures, successfully treated with a cast for 6 weeks. This study reports a rare and unusual case of Humero-radioulnar synostosis, in a patient with no family history of malformations.

## Image en medicine

La synostose huméro-radioulnaire est une déformation rare du membre supérieur. La synostose du coude est une malformation qui peut se produire entre n'importe lequel des os de cette articulation. La plus fréquente est la synostose radio-ulnaire. La synostose huméro-radioulnaire a été très peu rapportée dans la littérature. Nous rapportons le cas d'un homme de 32 ans, avec un état de phacomolie, consultant aux urgences, pour une douleur du membre supérieur gauche après un léger traumatisme de football. Le patient présentait également une craniosynostose de la suture lambdoïde. La radiographie du bras gauche a montré un humérus bifurqué, avec une déficience ulnaire, et des fractures radiales et ulnaires non déplacées, traitées avec succès par un plâtre pendant six semaines. Cette étude rapporte un cas rare et inhabituel de synostose huméro-radioulnaire, chez un patient sans antécédents familiaux de malformations.

**Figure 1:**
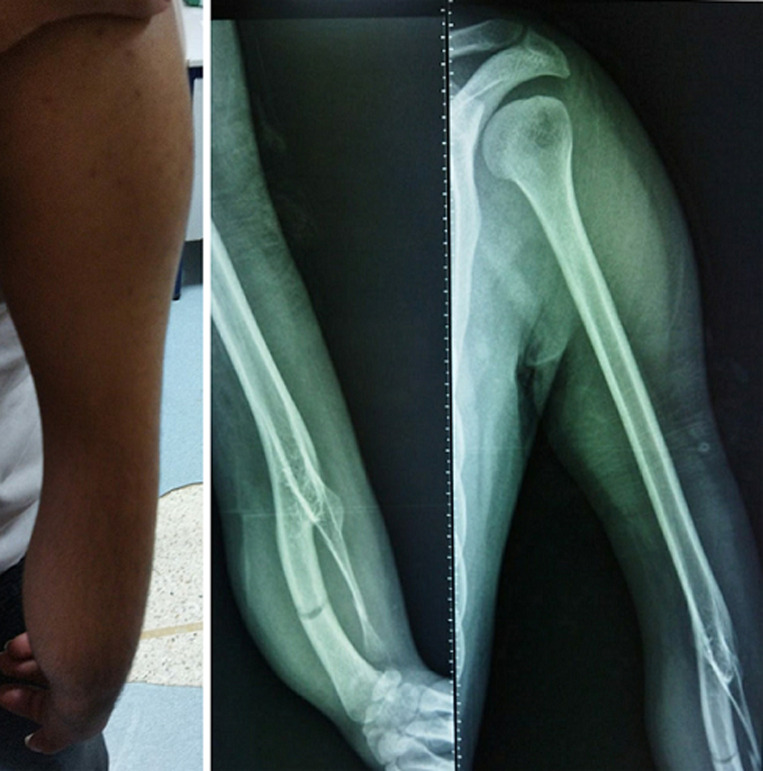
fracture des deux os de l'avant bras sur une synostose huméro-radioulnaire

